# Upadacitinib in refractory ulcerative colitis patients with high thromboembolic risk: two case reports

**DOI:** 10.3389/fimmu.2026.1716943

**Published:** 2026-03-03

**Authors:** Rui Mo, Shuxia Yu, Hongwei Xu, Jinghua Hao

**Affiliations:** Department of Gastroenterology, Shandong Provincial Hospital Affiliated to Shandong First Medical University, Jinan, Shandong, China

**Keywords:** case reports, rivaroxaban, thromboembolism, ulcerative colitis, upadacitinib

## Abstract

Upadacitinib is a novel selective Janus kinase (JAK) inhibitor approved for adults with ulcerative colitis (UC). While caution is advised when considering upadacitinib as a treatment option for patients with an increased risk of thrombosis. The current case report describes two UC patients with high thromboembolic risk successfully treated with upadacitinib and rivaroxaban. The two patients reported increased stool frequency, decreased stool consistency, abdominal pain and hematochezia, both of whom had experienced mesalazine, corticosteroids, vedolizumab and infliximab, all failed to induce clinical remission. The male patient had a history of deep vein thrombosis (DVT) in his left lower limb, and the female patient had thrombophlebitis in a branch of the cephalic vein on the left forearm. After multidisciplinary conference and thorough informed consent, upadacitinib and rivaroxaban were applied. Two months after the initiation of treatment, the patients’ symptoms and endoscopic findings improved significantly. No thromboembolic events or other adverse effects have been observed to date. Our case report suggests that concurrent use of anticoagulation with upadacitinib may represent a safe and valuable strategy for treating refractory UC with high thromboembolic risk.

## Introduction

Ulcerative colitis (UC) is a chronic inflammatory bowel disease (IBD) that is characterized by a spectrum of symptoms including abdominal pain, diarrhea, rectal bleeding, and weight loss. The aim of therapeutic management is to induce a rapid clinical response and to achieve clinical and endoscopic remission. Despite the increasing number of available therapeutic agents, 10-20% of patients still require proctocolectomy due to medically refractory disease (1).

Upadacitinib, a selective Janus kinase (JAK) inhibitor, has been licensed for treating adults with moderate-to-severe UC. Available data from randomized controlled trials (RCTs) showed that upadacitinib ranked highest for the induction of clinical remission and endoscopic improvement and was notably superior for these outcomes to all other small molecule drugs and biologics (2). However, upadacitinib must be used cautiously as it ranked highest when considering adverse events, especially regarding the potential risk of venous thromboembolism (VTE). The warnings issued by the Food and Drug Administration (FDA) and the European Medicines Agency highlight this risk (3, 4).

Post-marketing safety data for upadacitinib from global pharmacovigilance databases showed a disproportionately higher reporting rate of VTE compared to all other drugs, suggesting a potential safety signal warranting monitoring in clinical practice (5). As IBD is an established risk factor for VTE (6), it is imperative to not forget the importance of starting patients on thromboprophylaxis. However, no strategies have been proposed to mitigate the elevated VTE risk associated with upadacitinib in IBD patients. Here we present two cases of refractory UC patients with high thromboembolic risk successfully treated with concurrent use of upadacitinib and anticoagulation therapy.

## Case reports

**Figure 1:**
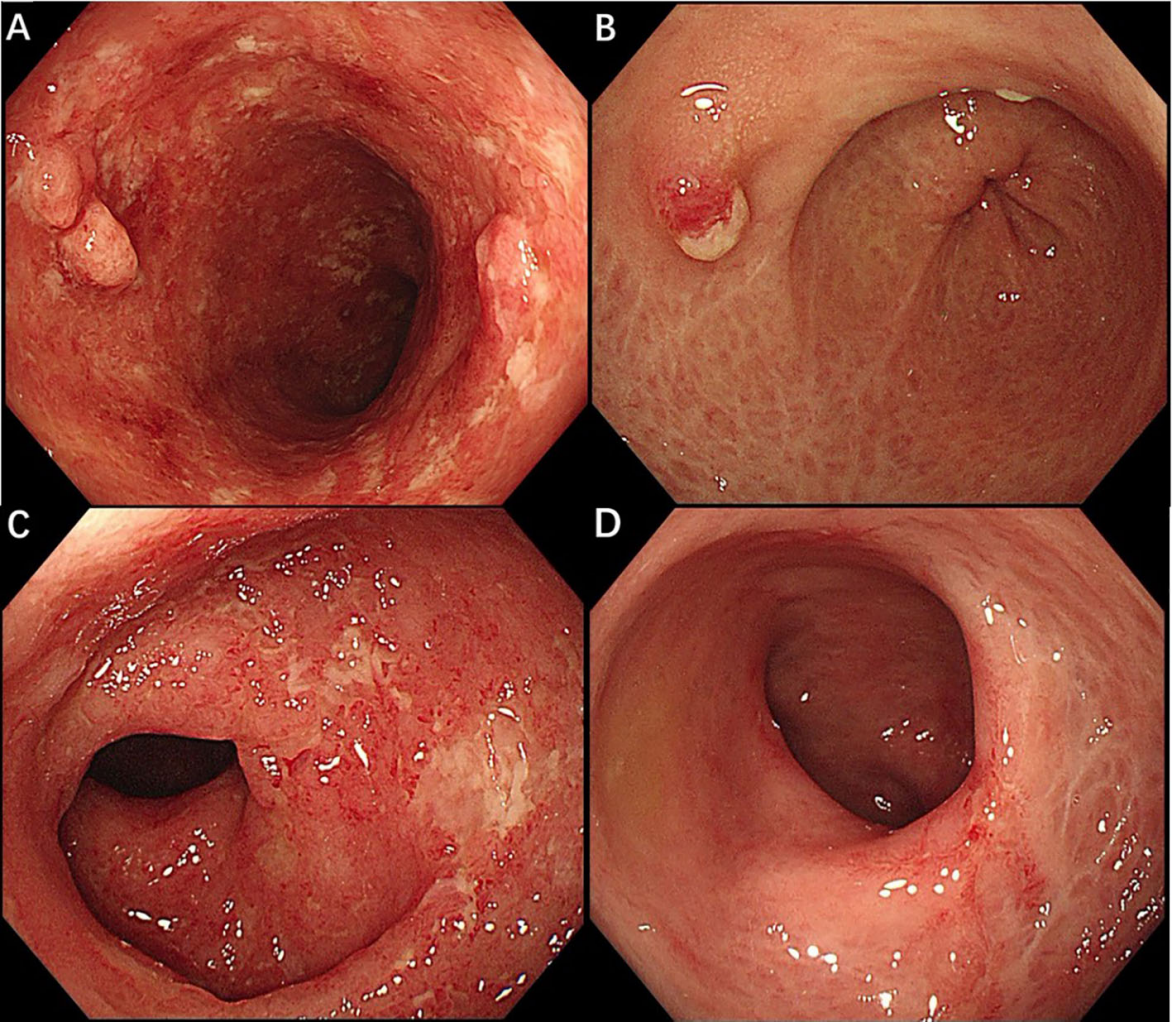
Endoscopic findings before and after upadacitinib treatment in patient 1 **(A, B)** and patient 2 **(C, D)**. **(A)** The sigmoid colon before starting upadacitinib, with ulcerations, loss of vascularity, pseudopolyps. **(B)** The sigmoid after 2 months of upadacitinib treatment showed healed mucosal ulcers with reticular scars. **(C)** The rectum before starting upadacitinib, endoscopy showed mucosal ulcers with a Mayo endoscopic score of 3. **(D)** At 2 months after upadacitinib, endoscopy demonstrated mucosal healing in the rectum.

The first patient, a 54-year-old man with a 10-year history of UC and no significant comorbidities, presented with abdominal pain and severe hematochezia. He was subjected to various treatments, including mesalazine, prednisone, vedolizumab and infliximab, with no response. He had a history of lower limb varicose veins and developed deep vein thrombosis (DVT) in the left lower extremity two years ago. Although the thrombosis improved with oral rivaroxaban, the hematochezia worsened. On admission, the D-dimer level was 0.62mg/L. Colonoscopy revealed left-sided colitis with a Mayo endoscopic score of 3. Upadacitinib 45mg daily was initiated for induction. Considering the high thrombotic risk, rivaroxaban was also prescribed at a dose of 10mg per day. Two months later, he achieved clinical remission and endoscopic improvement (**Figures 1A, B**). The D-dimer level normalized, and vascular ultrasound studies of the bilateral lower extremities showed no evidence of DVT.

The second patient is a 57-year-old woman with a 3-year history of ulcerative proctitis and no other chronic conditions. After receiving conventional treatments, including mesalazine and prednisone, the condition recurred repeatedly and eventually progressed to pancolitis. Furthermore, she showed primary nonresponse to vedolizumab and infliximab, and she refused to take azathioprine. The D-dimer level was 1.17mg/L. Of note, vascular ultrasound revealed thrombophlebitis in a branch of the cephalic vein on the left forearm. Due to the development of refractory UC, therapy was transitioned to upadacitinib 45 mg once daily, combined with rivaroxaban at a dose of 10mg per day following a multidisciplinary discussion involving gastroenterology and vascular surgery teams. Upon commencement of upadacitinib, her symptoms improved rapidly. She demonstrated endoscopic improvement (**Figures 1C, D**) without evidence of thrombophlebitis two months later, and her D-dimer level also normalized. Further details of changes in lipid parameters, hemoglobin, albumin, and C-reactive protein are presented in **Table 1**. We conducted monthly phone follow-ups to enhance adherence, during which no concomitant medications or adverse effects were recorded. Currently, both patients are receiving upadacitinib at a dose of 15mg per day for maintenance therapy, and rivaroxaban has been discontinued.

**Table 1:** Summary of laboratory test data of two refractory ulcerative colitis patients with high thromboembolic risk treated with upadacitinib.

Parameter	Patient1		Patient2	
	Pre-Upadacitinib	Post-Upadacitinib	Pre-Upadacitinib	Post-Upadacitinib
BMI(Kg/m2)	16.02	18.63	25.95	26.3
Hemoglobin(male 130-175g/L, female 115-150g/L)	123	121	86	104
Albumin(40-55g/L)	42	47.2	31.1	45.8
Hs-CRP(0-8mg/L)	1.4	0.42	17.2	1.88
ESR(0-20mm/h)	10	10	38	21
TG(<1.7mmol/L)	1.44	1.9	1.58	1.82
TC(<6.2mmol/L)	5.63	6.71	5.06	8.01
LDL-C(<3.37mmol/L)	3.37	4.23	3.32	4.89
HDL-C(1.04-1.55mmol/L)	1.42	1.86	0.96	2.18
D-dimer(0-0.5mg/L)	0.62	0.04	1.17	0.27

BMI, body mass index; Hs-CRP, high-sensitivity C-reactive protein; ESR, erythrocyte sedimentation rate; TG, total triglycerides; TC, total cholesterol; LDL-C, low-density lipoprotein cholesterol; HDL-C, high-density lipoprotein cholesterol.

## Discussion

JAK inhibition is a valuable new therapeutic strategy to treat UC by targeting a wide range of JAK-dependent cytokines. JAK inhibitors regulate a more diverse array of genes than anti-tumor necrosis factor-α blockers, which may explain why these two patients, both with a history of loss of response to vedolizumab or infliximab, responded to upadacitinib. In addition, the oral route of administration, the fast onset of action, and the short half-life are key advantages of JAK inhibitors.

Currently, tofacitinib, filgotinib and upadacitinib are JAK inhibitors approved for UC. Tofacitinib is a pan-JAK inhibitor, mostly targeting JAK1 and JAK3, and is the first JAK inhibitor approved for treating UC. In 2021, the FDA updated the record of adverse effects linked to the use of tofacitinib, adding to it the increased risk of blood clots and of death. Health care professionals should educate patients thoroughly about these detrimental events and advise them to carefully weigh the risks and benefits when considering tofacitinib and avoid using tofacitinib in patients who may have a higher risk of thrombosis. This was done after revealing a higher risk of acquiring the aforementioned risks in patients receiving tofacitinib (7). Upadacitinib is a selective small-molecule JAK1 inhibitor, comparative analyses showed that upadacitinib-treated patients demonstrated higher efficacy and a lower risk of discontinuation than patients treated with other JAK inhibitors (8). The safety concerns of upadacitinib were not studied as comprehensively as those of tofacitinib. However, since upadacitinib shares the same mechanism of action as tofacitinib and descends from the same drug class, the FDA deemed it necessary to include upadacitinib in this update presuming that it has the same adverse effects as tofacitinib.

Patients with IBD have an increased risk of thrombosis, which has been reported to be 2- to 3-fold higher than that of patients without IBD and is exacerbated during times of disease flare (6). The Caprini scoring system provides a consistent, thorough, and efficacious method for risk stratification of VTE. The total Caprini risk score was 5 for both patients, which could be categorized as high-risk for venous thrombosis (9). Moreover, for patients with a history of VTE prior to JAK inhibitor initiation, there is an elevated risk (9%) of recurrent VTE (10). Notably, a treatment approach of beginning therapeutic anticoagulation along with a JAK inhibitor may improve the safety of JAK inhibitor use.

Studies showed that symptomatic relief from UC was evident as early as 1 to 3 days after starting treatment with upadacitinib (11). Similarly, our patients reported improvement in their symptoms within 3 days after starting on upadacitinib 45 mg orally once daily. After two months of upadacitinib treatment, lipid levels were increased in both patients. Nevertheless, no association between the changed lipid profile and cardiovascular events has been established in previous clinical studies (12). Following the achievement of endoscopic remission, the upadacitinib dose was stepped down to 30 mg daily for two weeks, then to 15 mg daily for maintenance therapy, rivaroxaban was also discontinued at that time to minimize the bleeding risk. Further clinical studies are required to evaluate the rationale behind this proposed strategy. Despite this, the concurrent use of upadacitinib with anticoagulation appears to be a promising and safe option for patients with refractory UC and high risk for thromboembolism.

## Data Availability

The raw data supporting the conclusions of this article will be made available by the authors, without undue reservation.
